# Experience of Rwanda on COVID-19 Case Management: From Uncertainties to the Era of Neutralizing Monoclonal Antibodies

**DOI:** 10.3390/ijerph19031023

**Published:** 2022-01-18

**Authors:** Menelas Nkeshimana, Deborah Igiraneza, David Turatsinze, Otto Niyonsenga, Deborah Abimana, Cyprien Iradukunda, Emmanuel Bizimana, Jean Muragizi, Lise Mumporeze, Laurent Lussungu, Hackim Mugisha, Elizabeth Mgamb, Noella Bigirimana, Edison Rwagasore, Swaibu Gatare, Hassan Mugabo, Olivier Nsekuye, Muhammed Semakula, Augustin Sendegeya, Ephraim Rurangwa, Edgar Kalimba, Sanctus Musafiri, Corneille Ntihabose, Eric Seruyange, Charlotte Bavuma, Theogene Twagirumugabe, Daniel Nyamwasa, Sabin Nsanzimana

**Affiliations:** 1 Rwanda Joint Task Force for COVID-19, Case Management Sub-Cell, Kigali 84, Rwanda; igidebbie@gmail.com (D.I.); dturatsinzeh2001@yahoo.fr (D.T.); cyira55@gmail.com (C.I.); emmyone00@gmail.com (E.B.); jmuragizi@gmail.com (J.M.); mugishahackim@gmail.com (H.M.); 2 Clinical Services Division, Centre Hospitalier Universitaire de Kigali, Kigali 655, Rwanda; musanct@gmail.com (S.M.); charlottebavuma5@gmail.com (C.B.); 3 School of Medicine and Pharmacy, College of Medicine and Health Sciences, University of Rwanda, Kigali 4285, Rwanda; niyot10@gmail.com (O.N.); lise.mumporeze@kfhkigali.com (L.M.); ericseruyange@gmail.com (E.S.); twagirumugabe@gmail.com (T.T.); 4 Clinical Services Division, Centre Hospitalier Universitaire de Butare, Huye 254, Rwanda; augdr2002@gmail.com; 5 Nyarugenge District Hospital, Kigali 5634, Rwanda; aderamia12@gmail.com; 6 King Faisal Hospital, Kigali 2534, Rwanda; edgar.kalimba@kfhkigali.com; 7 Hôpital La Croix du Sud, Kigali 3377, Rwanda; lussungu@yahoo.fr; 8 World Health Organization, Asmara 5561, Eritrea; mgambe@who.int; 9 Rwanda Biomedical Center, Kigali 7162, Rwanda; noella.bigirimana@rbc.gov.rw (N.B.); edison.rwagasore@rbc.gov.rw (E.R.); gatare.swaibu@rbc.gov.rw (S.G.); hassan.mugabo@rbc.gov.rw (H.M.); nsolly03@gmail.com (O.N.); semakulam@gmail.com (M.S.); 10 Rwanda Military Hospital, Kigali 3377, Rwanda; comdtrmh@minadef.gov.rw; 11 Rwanda Ministry of Health, Kigali 84, Rwanda; corneille.ntihabose@moh.gov.rw (C.N.); dnyamwasa@gmail.com (D.N.); 12 Kacyiru Police Hospital, Kigali 6304, Rwanda

**Keywords:** COVID-19, interventions, outcomes, monoclonal antibodies, guidelines, Rwanda

## Abstract

The management of COVID-19 in Rwanda has been dynamic, and the use of COVID-19 therapeutics has gradually been updated based on scientific discoveries. The treatment for COVID-19 remained patient-centered and entirely state-sponsored during the first and second waves. From the time of identification of the index case in March 2020 up to August 2021, three versions of the clinical management guidelines were developed, with the aim of ensuring that the COVID-19 patients treated in Rwanda were receiving care based on the most recent therapeutic discoveries. As the case load increased and imposed imminent heavy burdens on the healthcare system, a smooth transition was made to enable that the asymptomatic and mild COVID-19 cases could continue to be closely observed and managed while they remained in their homes. The care provided to patients requiring facility-based interventions mainly focused on the provision of anti-inflammatory drugs, anticoagulation, broad-spectrum antibiotic therapy, management of hyperglycemia and the provision of therapeutics with a direct antiviral effect such as favipiravir and neutralizing monoclonal antibodies. The time to viral clearance was observed to be shortest among eligible patients treated with neutralizing monoclonal antibodies (bamlanivimab). Moving forward, as we strive to continue detecting COVID-19 cases as early as possible, and promptly initiate supportive interventions, the use of neutralizing monoclonal antibodies constitutes an attractive and cost-effective therapeutic approach. If this approach is used strategically along with other measures in place (i.e., COVID-19 vaccine roll out, etc.), it will enable us to bring this global battle against the COVID-19 pandemic under full control and with a low case fatality rate.

## 1. Introduction

Since the World Health Organization (WHO) declared the 2019 novel coronavirus outbreak a public health emergency of international concern on 30 January 2020 [1], with the disease thereafter named COVID-19, the pandemic has brought unprecedented social and economic disruptions globally, while numbers of cases and deaths have soared [2]. COVID-19 is caused by the severe acute respiratory syndrome coronavirus 2 (SARS-CoV-2) belonging to the family of Coronaviridae [3]. At the time of this writing, around 173 million infected cases had been identified across the globe, including nearly 4 million of deaths [4]. The disease continues to present a major public health challenge to the entire world, with an increasing number of cases in several countries. To date, the United States of America, Brazil, and India have registered the highest number of cases while Africa remains among the least affected continents.

Rwanda reported the first case of COVID-19 on 14 March 2020, and since then, of 1,466,941 tests performed, 27,211 individuals have been confirmed positive and 1.3% succumbed to the disease as of 9 June 2021 [5]. Following the WHO declaration of the pandemic, the Government of Rwanda immediately adopted and implemented several public health and social measures aiming at slowing down the spread of the pandemic. These measures included countrywide lockdown, border closure, banning unnecessary travel, prohibiting public gatherings, the use of face masks in public places, the closure of schools, churches and non-essential services and setting up hand hygiene facilities in all public places. The Rwanda Joint Task Force for COVID-19 was established to coordinate and monitor the implementation of these measures. Pre-existing rapid response teams, including the case management workforce, which were initially set up to tackle the spread of Ebola, are now operational from the lowest level of the Rwanda health structure, i.e., from health centers, to the national referral hospitals. Case management and infection control teams as part of the task force are mandated to coordinate the clinical aspects of the disease at all levels of the healthcare system.

Similarly to every other country, initially Rwanda has faced COVID-19 management challenges such as inadequate infrastructure to accommodate all the required infection prevention control (IPC) measures, lack of oxygen, and unprepared and limited numbers of trained staff. Although global efforts towards specific therapies for the disease are ongoing, they were not well coordinated at the beginning of the pandemic. However, no conventional effective treatment has been validated thus far. Currently, different therapeutic approaches targeting the main phases of COVID-19 pathogenesis, namely, the fusion and entry of the virus via the host cell receptors, viral replication and the immuno-inflammatory phase known as a “cytokine storm”, are subject to clinical trials worldwide [6,7,8]. The therapeutic approaches for COVID-19 have been changing, guided by the evolution of knowledge on the disease physiopathology to develop evidence-based guidelines for its management [9]. Therefore, this has also evolved alongside the discovery of potential effective medications.

Rwanda is one of few countries that have quickly adopted and implemented early and strong mitigation measures, and progressive capacity building to fight COVID-19. The magnitude of the pandemic has been alleviated by multidisciplinary interventions materializing in teamwork, good leadership, flexibility and adaptation of therapeutic guidelines based on the evolution of knowledge and continuous local assessments of pandemic trends. It is in this context that we aim to share the experience of Rwanda on the treatment of COVID-19 that has demonstrated positive outcomes during the first and second surges.

## 2. Objectives

1. To document the evolution of COVID-19 case management interventions since the beginning of the pandemic;
2. To highlight lessons learnt in COVID-19 case management and the way forward as we promote the culture of experience sharing across the world for mutual learning and support in this global fight against the COVID-19 pandemic.

## 3. Methods

### 3.1. Study Design and Source of Information

This was a retrospective descriptive study on the experience on COVID-19 case management in Rwanda. The information was drawn from the standard operating procedures (SOPs) issued in March 2020 and COVID-19 clinical management guidelines for Rwanda. The three consecutive versions of the guidelines were considered to gather information on the use of different therapies to treat identified cases. Patient medical records and health management information systems (HMISs) were also used to extract socio-demographic and therapeutic outcome data.

Proportions of patients who underwent care in the main treatment areas (home or health facilities) were plotted on curves along the time since the notification of the first case up to March 2021 by using the R software version R-4.1.0 (R Core Team (2018), Vienna, Austria). From the data accessed, the analysis was made using 2696 patients who received symptomatic care alone, 394 who received favipiravir and 64 who were infused with neutralizing monoclonal antibodies. The latter were introduced into the national guidelines at a later stage, with stricter indications to be used for patients at highest risk of developing severe disease because the available doses were not sufficient to cover all the patients who would have benefited from this therapy. We used proportional Cox hazard regression to compare the impacts of three different therapeutic interventions on the time to viral clearance.

### 3.2. Variables’ Definition

#### 3.2.1. Demographic Variables

Age was defined as the reported completed years, months or days of life for children under one year of age based on the date of birth of the patient as captured in the patient’s medical records.

Gender: as socially constructed in Rwanda, it was self-reported as “male” or “female”.

Residence (province) refers to the administrative residential localities: Kigali city, Northern Province, Southern Province, Eastern Province and Western Province.

#### 3.2.2. Clinical Variables

Diagnostic modality: two testing modalities have been in use in Rwanda, including real-time reverse transcriptase polymerase chain reaction (rt-PCR) and the antigen-based rapid diagnostic test (RDT). Individuals who tested positive on either test modality were considered as confirmed positive.

Diagnosis date refers to the date of collection of the sample which turned positive.

Admission date refers to the day on which the patient was registered and admitted to the inpatient treatment ward at the hospital isolation center or treatment center.

Treatment center refers to the clinical spaces, separated from the pre-existing health facilities, created or repurposed to take care of the patients with a diagnosis of COVID-19 to ensure that nosocomial transmission is prevented.

Comorbidities are other concurrent pre-existing or new-onset medical conditions (diabetes mellitus, hypertension, renal disease, asthma, cancer, HIV infection, etc.) with which the patient is diagnosed, and may have negative impacts on COVID-19 outcomes.

Type of treatment refers to the categories of the main therapeutic approaches used in the treatment of COVID-19 in Rwanda. This includes symptomatic therapy and antiviral drugs such as favipiravir or bamlanivimab.

Home-Based Care refers to the self-care of patients in their homes with follow ups. This category of patients is either asymptomatic or with a mild COVID-19 infection with no risk factors for disease progression, as recommended by the WHO.

#### 3.2.3. Outcomes Variables

Time to viral clearance was defined as the time between the first positive test for COVID-19 and the time to negative test performed for the purpose of control after treatment in either modality of care. During the first surge ranging from March to September 2020, a second negative test was performed within 24 h after the first negative. During the second surge, from September 2020 to February 2021, the time to clearance was changed to the time from the first positive test to the first negative test.

Length of hospital stay was the duration of stay in the treatment center or health facility from the date of admission to discharge, irrespective of the results of the control test.

COVID-19-related death is death after testing positive for COVID-19 and death from complications of COVID-19 such as ARDS, lung fibrosis, coagulation disorders, etc.

## 4. Results

**Figure 1:**
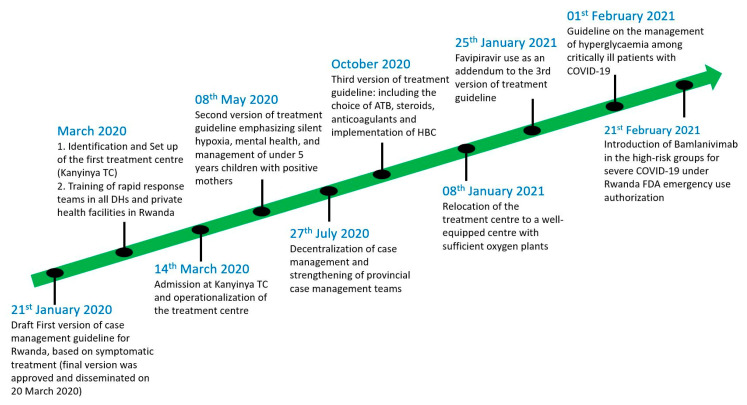
Timeline of COVID-19 case management interventions.

Since almost one month before the first confirmed COVID-19 case in Rwanda on 14 March 2020, different strategies for case management have been adopted and implemented. Treatment centers and quarantine sites were created, and some health facilities and schools were repurposed to ensure that the non-mixing international protocols were applied. Guidelines for the supportive, symptomatic and specific treatment of COVID-19 cases were developed and continuously updated (Figure 1).

### 4.1. Set up of Quarantine, Isolation and Treatment Facilities for COVID-19 across the Country

The experience of Rwanda from the time of preparedness phase to the full-scale of response is a living testimony; such a global health threat requires a well-coordinated global response.

The COVID-19 case management process was initiated during the pre-epidemic period with the adaptation of the already existing Ebola virus preparedness plan and facilities [10,11]. These included isolation centers within referral hospitals and district hospitals, especially those bordering the Democratic Republic of Congo (DRC). The patient flow and screening process was based on case definition [12]. Instructed by the Ministry of Health, all health facilities operating in Rwanda were requested to avail at least two isolation rooms for COVID-19 patients. Additionally, some existing health facilities were completely transformed into COVID-19 treatment centers, demonstrating the flexibility of the Rwandan health system and expandability of the clinical spaces allocated to COVID-19. A typical example of a repurposed facility is Kanyinya Health Center, initially constructed in 2013 and then fully transformed into a COVID-19 treatment center in March 2020 (Figure 1).

The center has an 83-bed capacity, including 8 beds dedicated for intensive care unit (ICU) cases. All patients with confirmed COVID-19 were taken to the designated COVID-19 facilities irrespective of the clinical status or severity of the disease. Individuals under investigation for potential SARS-CoV-2 infection, and those who were in contact with the known positive cases, were also systematically taken to the holding facilities (also known as quarantine sites). It is worth noting that all these services were offered free of charge, in order to ease access to these facilities. As the number of cases was rising and health workers from different corners of the country were trained, the number of treatment centers increased accordingly, aligning well with the national decentralization plan (Figure 1).

**Figure 2:**
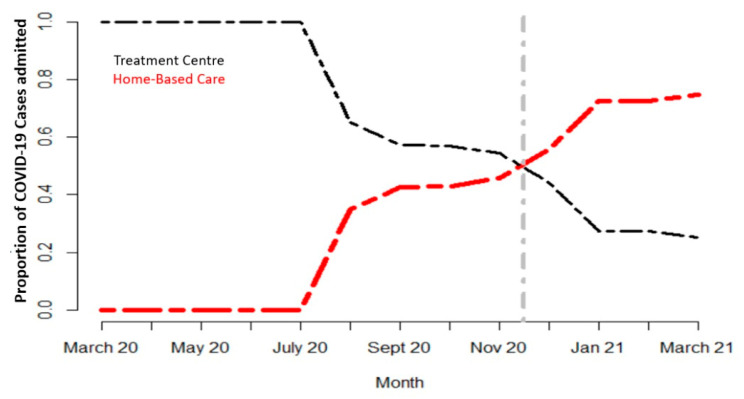
Trend in proportions of COVID-19 cases in HBC and treatment centers.

The number of people needing advanced clinical care remained low (less than 10% of total confirmed cases) compared with the total number of individuals infected with SARS-CoV-2. This paved the way for discussions at the strategic level on the cost-effectiveness of modalities of treatment. A pilot project was launched in some districts of Rwanda with a high number of cases, to assess the feasibility of the home-based care (HBC) approach. As the HBC scheme took shape, the number of patients in treatment centers drastically decreased (Figure 2). It was found safe and its acceptability by the community was quite good as long as social protection motives could follow, such as the distribution of food and other daily life necessities to patients in need.

From November to December 2020, as the second wave spread faster, a fivefold increase in the number of deaths was recorded compared with that of the first wave, culminating in over 200 deaths from December 2020 to February 2021 [5].

**Figure 3:**
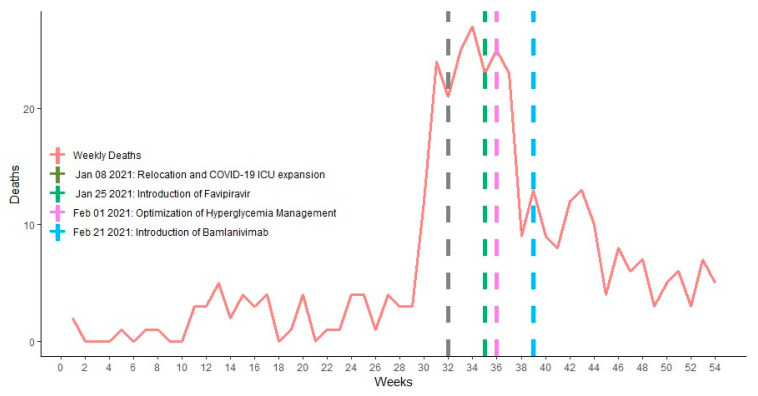
Major case management therapeutic interventions during COVID-19 deaths surge.

From the death audit conducted in early January 2021, there was a recommendation to relocate all the COVID-19 patients in need of advanced clinical care to the newly furnished 136-bed hospital equipped with piped oxygen (Figure 3). This hospital was then staffed with most knowledgeable trained clinicians with regular mentorship by experienced professionals.

In the following months, the number of critically ill cases gradually decreased (Figure 3) in some areas and a stronger surveillance mechanism facilitated the implementation of the “4 Early” strategy: “early suspicion, early testing/diagnosis, early initiation of treatment and an early referral to a higher facility”, if any of therapeutic items were missing from the ground facility. At the time of writing, facilities to accommodate critically ill COVID-19 patients are now being expanded to make them accessible in different corners of the country.

Amid the second COVID-19 wave in Rwanda, as several districts were threatened with the imminent risk of community transmission, there was a policy shift in clinical management that required a more decentralized care approach. This consisted of supporting the provincial case management teams with the aim of reducing the number of referrals from remote areas to be treated in Kigali (Figure 1). This was further supported by the training programs that were rolled out by several professional societies (i.e., Rwanda Medical Association etc.), in order to increase both the confidence and skills of healthcare workers treating COVID-19 patients.

As the number of critically ill COVID-19 patients peaked, the clinical will and experience accompanied the healthcare providers in their day-to-day challenges, and provided clinical guidelines addenda to the management of hyperglycemia and safe sedation for COVID-19 patients undergoing mechanical ventilation in the high-dependency and intensive care units (Figure 3).

### 4.2. Faith in Science: The Basis of Evidence-Based Case Management in Rwanda

The national case management team, part of the Scientific Advisory Group in Rwanda, created in March 2020, has closely supervised the creation, equipping and staffing of all COVID-19 treatment centers. The group continues to be engaged at the strategic level to ensure that the guidelines in use are well positioned to respond to several aspects of the pandemic, both at the community level and in health facilities.

At the onset of the pandemic in Rwanda, common practice in all existing health facilities’ clinical management guidelines was to provide symptomatic treatments because little was still known about the pathophysiology behind the progression to acute respiratory distress syndrome (ARDS) in COVID-19 patients and the use of antiviral therapies was not supported with strong evidence. This was referred to within the first version of national case management guidelines for Rwanda (Figure 1) [13]. This version, issued on 20 March 2020 and referring mainly to the available information from the WHO, was focused on the optimization of supportive and symptomatic treatment, and it recommended the early detection of subjects with severe acute respiratory infection (SARI) associated with COVID-19 and hitherto the subsequent prioritization of care. The triage considered six levels of disease, which included mild illness with uncomplicated upper respiratory tract infection, pneumonia not requiring oxygen therapy, severe pneumonia associated with signs of severe respiratory distress, acute respiratory distress syndrome (ARDS) with features of respiratory failure, sepsis with life-threatening organ dysfunction and septic shock.

The appropriate treatment was also described in relation to the severity of the disease. Asymptomatic subjects, who were reported to be potentially contagious, were isolated to contain virus transmission. Those with mild symptoms were treated for symptomatic relief, with antipyretics and cough mixtures. Although there was no indication for hospitalization, those cases were kept in health facilities and in repurposed non-traditional settings to contain and mitigate transmission. Furthermore, patients were counselled on signs of severity for early evacuation to an appropriate facility equipped with an intensive care unit. For severe cases of COVID-19, it was recommended to provide a supplemental oxygen therapy to patients with SARI and respiratory distress, hypoxemia or shock. The target was to reach SpO2 > 94% for patients receiving 5 L/min, SpO2 ≥ 93% during resuscitation with 10–15 L/min of oxygen requirements. All areas caring for patients with SARI were equipped with pulse oximeters and functioning oxygen systems. In addition, bacterial superinfections were treated with empirical antimicrobials against all likely pathogens causing SARI. Cases progressing to acute respiratory distress syndrome (ARDS) despite standard oxygen therapy were intubated and assisted with mechanical ventilation. This version of the guideline prohibited the use of systemic corticosteroids because it was suspected to have no survival benefit and even be potentially harmful in terms of delaying viral clearance for instance. Only inhaled steroids, hydroxychloroquine, chloroquine phosphate, remdesivir and lopinavir/ritonavir were recommended drugs for COVID-19 infection in Rwanda as trials. However, these products were not yet available for this purpose.

At that time, the patient was discharged following a clinical improvement for more than 48 h, having stayed in the treatment center for a minimum of 14 days regardless of how soon the patient had improved. Two negative results of COVID-19 rt-PCR, 24 h apart, and the willingness to remain in self-isolation at their homes for an additional two weeks from the day of discharge were among the criteria for discharge from treatment centers.

As further clinical trials emerged, the initiation of anticoagulants and dexamethasone became an acceptable reality, which is well referenced in the second and third versions of COVID-19 management guidelines for Rwanda [14,15]. The care for the pediatric population, pregnant women, psychological support to positive cases and the non-separation of children less than 5 years of age from their positive mothers are other highlights of these revised versions.

As part of the “hit hard, hit early principle” in COVID-19 management, based on clinical trials performed in UAE, China, Russia and Japan [16], favipiravir was rolled out for use as an anti-SARS-CoV-2 therapy in Rwanda since January 2021 (Figure 3) to decrease the number of patients needing ICUs. As of today, in the same global move to determine what would be effective to protect people at high risk of disease progression to severe COVID-19, the use of neutralizing monoclonal antibodies is gaining ground in Rwanda, especially in the treatment of population at highest risk of developing severe COVID-19 (Figure 3).

### 4.3. Impact of Therapeutic Strategies on the Duration of Virus Shedding

When analyzed for the time to viral clearance, patients with COVID-19 who only received a symptomatic treatment tended to clear the virus more slowly than those who received favipiravir (HR, 95% CI: 1.97 [1.74–2.23]; *p* < 0.001). Patients who benefited from the single 700 mg dose of bamlanivimab cleared the virus even faster than both those treated with favipiravir and those with symptomatic treatments (HR, 95% CI: 5.56 [4.07–7.58]; *p* < 0.001) (Table 1).

**Figure 4:**
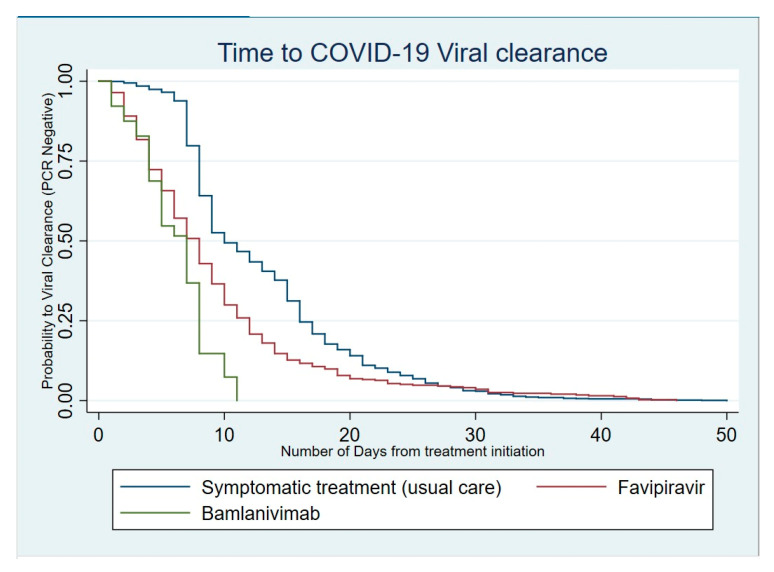
Kaplan–Meier curve for viral clearance according to the therapeutic intervention.

In fact, almost all patients who received the neutralizing monoclonal antibody therapeutic approach cleared the virus within 11 days (Figure 4).

Similarly, the majority of patients (75%) treated with favipiravir also cleared the virus within 11 days. However, a proportion close to 10% shed the virus for more than 20 days.

## 5. Discussion

Since the first COVID-19 case detection in Rwanda, treatment guidelines were adapted to the evolution of knowledge and the epidemiological trend of the pandemic. The first and second COVID-19 clinical guidelines for Rwanda recommended admission to health facilities of all confirmed cases for symptomatic and supportive treatment. The ultimate goal to admit and isolate every positive case was to limit the spread of the virus within the country as much as possible while planning the home-based care of asymptomatic cases. This approach was mainly guided by WHO clinical guidelines issued in January 2020 [17,18]. During this period, characterized by much uncertainty and lack of evidence-based effective drug therapy, Rwanda did not record deaths, which was mainly due to the fact that most cases were imported, with less transmission to vulnerable groups in the community, facilitated by strong and early effective public health measures.

With an increasing number of cases, especially during the second wave of the pandemic, and scientific insights, a pressing need to revise the case management strategies was felt.

As recommended by the WHO and the national Scientific Advisory Group, Rwanda moved to the management in the community (home-based care) of cases with no or mild symptoms of COVID-19 and without any comorbidity that could predispose to a progression to severe forms of the disease. This approach is an economically sound and clinically reasonable strategy even in resource-variable settings, as per a study performed by Barasa et al. in Kenya [19,20]. In fact, evidence has shown that the majority of individuals receiving home-based care who progress to severe COVID-19 are those with advanced age and/or with comorbidities [21,22].

The risk of progressing to life-threatening conditions of COVID-19 needs to be considered when deciding on the best therapeutic strategies. Lack of enough evidence on the best pharmacotherapy delayed the decision to treat, leaving the lead to medical reasoning. One of the strategies adopted by Rwanda was the introduction of new repurposed therapies that were found to be efficient, safe and with potential to limit the progression of the disease. Favipiravir, which is an antiviral that was initially aimed to manage the influenza pandemic in Japan in 2014, is reported as an efficacious drug that reduces the SARS-CoV-2 replication phase, limiting the progression to severe COVID-19 per se through a faster clearance of the virus [23,24,25].

In our experience, we observed a relatively faster viral clearance compared with symptomatic and supportive care. More than three-quarters of those who were receiving favipiravir cleared the virus within 11 days. This short time to clearance has been reported in other studies [16,24]. Moreover, the case fatality rate (CFR) may have been reduced but we could not analyze the specific CFR adjusted for age in comparison with those who did not receive favipiravir. This needs to be considered for a specific study. In this perspective of reduced duration of virus shedding, the reduction in transmissibility to contacts is probably another potential benefit that needs further exploration in the Rwandan context.

Further to the introduction of favipiravir, Rwanda has recently acquired bamlanivimab infusion, which was introduced in February 2021. A large majority of the infused patients in Rwanda had negative PCR tests before 11 days. Bamlanivimab is one of the neutralizing monoclonal antibodies (mAbs) that has been authorized by the U.S. Food and Drug Administration (FDA) for emergency use in the treatment of patients with mild to moderate COVID-19 of 12 years of age and older, weighing at least 40 kg, qualified as subjects at high risk for severe COVID-19 [26]. It is reported to not only have no benefits, but also be associated with worse clinical outcomes in severe COVID-19, thus recommended only for asymptomatic, mild and moderate COVID-19 [26,27]. Thus far, we have not recorded any acute infusion side effect or incident, similarly to other large studies. However, the effect of bamlanivimab in monotherapy is currently reported to show no superiority to a placebo in terms of viral load reduction, but it remains effective in terms of reduction in hospitalization needs: a proxy of the progression to severe COVID-19 [28]. Recently, the U.S. FDA revoked the authorization for its emergency use as monotherapy, and rather recommended a combined therapy due to recent trial results showing the risk of drug resistance and neutral effect on the reduction in viral load [28,29].

Although the effectiveness of bamlanivimab alone to reduce the viral load in COVID-19 patients is now being questionable, mainly due to emergence of induced resistant mutants, it remains recommended when associated with etesevimab, another anti-spike monoclonal antibody binding to an epitope of SARS-CoV-2 target cell receptor, different from but complementary to that of bamlanivimab. With this association, a faster viral clearance is regained [30]. Therefore, continued surveillance for the early detection of emergent mutations that may annihilate the effect of this therapy in Rwanda is recommended. Moreover, the introduction of etesevimab in Rwanda for use in combination with bamlanivimab also deserves special consideration. This may be a reasonable approach to reduce the number of patients who may progress to severe COVID-19, die from it and overwhelm the health system in the expectative period to an effective vaccination coverage for COVID-19 in the country, which is yet to come [31].

This use of monoclonal antibodies for COVID-19 management in patients at high risk of progression to severe disease is a reasonable investment. One could question this cost-effectiveness in resource variable settings by looking at the prohibitive cost of this treatment. However, it is notable to keep in mind money savable from cases who may be protected from deteriorating to the point where they needed ICU admission with lengthy ventilator support and high oxygen supplementation, which generally results in death or severe chronic impaired respiratory function for survivors [19]. The majority of patients who require ICU care across Africa have generally succumbed from the disease, and Rwanda, even recognized as successful in controlling COVID-19, is no exception from this unfortunate observation during the second wave [32].

The data from clinical trials have shown that the neutralizing monoclonal antibodies can reduce disease progression by a rate which varies between 54% and 85% [33]. As we move forward, we are hoping to be able to carry out comparative studies across different countries using repurposed drugs such as favipiravir and the novel therapeutics in the category of neutralizing monoclonal antibodies which are not yet expanded due to their affordability. We believe that this paper, which articulates the positive signal of monoclonal antibody efficacy in a Rwandan cohort of patients with COVID-19, will serve to advocate availing these novel therapeutics to sub-Saharan Africa in a both timely and affordable manner. As of today, a limited number of countries have direct access to neutralizing monoclonal antibodies through initiatives that promote the equitable share of drugs (i.e., The COVID-19 Therapeutics Accelerator), and more is to be expected as we navigate through this prolonged pandemic.

## 6. Conclusions

As the number of COVID-19 cases continued to rise, Rwanda has progressively introduced and adjusted its treatment strategies to save the lives of infected people and to protect its health system from being overwhelmed by huge numbers of COVID-19 cases. Symptomatic and supportive treatments along with close monitoring of severity changes in home-based care constitute a good approach that should be strengthened. Antiviral treatment or combined monoclonal antibodies should be maintained for those at high risk of progressing to severe COVID-19. A revisiting of existing strategies to identify opportunities to scale them up to combat potential upcoming waves is key to keeping pace with the success observed thus far.

To the best of our knowledge, this is the first paper that describes the experience on the use of neutralizing monoclonal antibodies in sub-Saharan Africa. Although there are a number of limitations to this study, including details related to patients’ characteristics and comorbidities, we believe that this paper will serve to disseminate our positive signal that the implementation of novel COVID-19 therapeutics roll-out is not only feasible and safe, but has also saved the lives of many people in Rwanda. We recommend this equity agenda to be developed further, taken farther and much faster across the world.

## Figures and Tables

**Table 1:** Analysis of the time to viral clearance with adjustments for age and gender.

	Mean of Length of Hospital Stay	Mortality Rate	Time to Viral Clearance Median (IQR)	HR	95% CI	*p*-Value
**Treatment groups**						
Symptomatic treatment	9.4		6 (6–10)	Ref		
Favipiravir	9.1		7 (4–11)	1.97	(1.74–2.23)	0.000
Bamlanivimab	5.4	3/67 (4.4%)	6 (4–9)	5.56	(4.07–7.58)	0.000
**Sex**						
Female	10.0		6 (6–14)	Ref		
Male	9.0		6 (6–9)	1.15	(1.04–1.27)	0.007
Age				0.996	(0.993–0.999)	0.012

## Data Availability

The data that support the findings of this study are available upon request to the corresponding authors.

## References

[1] Cucinotta D., Vanelli M. (2020). WHO Declares COVID-19 a Pandemic. Acta Biomed..

[2] Policy Responses to COVID19. IMF. https://www.imf.org/en/Topics/imf-and-covid19/Policy-Responses-to-COVID-19.

[3] Severe Acute Respiratory Syndrome Coronavirus-2 (SARS-CoV-2). https://www.ncbi.nlm.nih.gov/pmc/articles/PMC7182166/.

[4] World Health Organization Coronavirus (COVID-19) Events as They Happen. https://www.who.int/emergencies/diseases/novel-coronavirus-2019/events-as-they-happen.

[5] Rwanda Biomedical Centre Situation Report on COVID-19. https://www.rbc.gov.rw/index.php?id=717&L=0.

[6] Interleukin-6 Receptor Antagonists in Critically Ill Patients with COVID-19 | NEJM. https://www.nejm.org/doi/full/10.1056/NEJMoa2100433.

[7] The RECOVERY Collaborative Group (2021). Dexamethasone in Hospitalized Patients with COVID-19. N. Engl. J. Med..

[8] Information on COVID-19 Treatment, Prevention and Research. COVID-19 Treatment Guidelines. https://www.covid19treatmentguidelines.nih.gov/.

[9] Therapeutic Management. COVID-19 Treatment Guidelines. https://www.covid19treatmentguidelines.nih.gov/therapeutic-management/.

[10] WHO Applauds Rwanda’s Ebola Preparedness Efforts. https://www.who.int/news/item/24-07-2019-who-applauds-rwanda-s-ebola-preparedness-efforts.

[11] Rwanda Uses Ebola Experience to Combat COVID-19. https://www.aa.com.tr/en/africa/rwanda-uses-ebola-experience-to-combat-covid-19/1813902.

[12] WHO COVID-19 Case Definition. https://www.who.int/publications-detail-redirect/WHO-2019-nCoV-Surveillance_Case_Definition-2020.2.

[13] Rwanda Ministry of Health, Rwanda Biomedical Center (2020). COVID-19 Clinical Guideline for Rwanda.

[14] Rwanda Ministry of Health, Rwanda Biomedical Center (2020). COVID-19 Clinical Guideline for Rwanda.

[15] COVID-19 Clinical Management Guidelines. https://www.rbc.gov.rw/fileadmin/user_upload/guide/Guidelines/COVID-19%20Clinical%20Managment%20guidelines.pdf.

[16] Joshi S., Parkar J., Ansari A., Vora A., Talwar D., Tiwaskar M., Patil S., Barkate H. (2021). Role of favipiravir in the treatment of COVID-19. Int. J. Infect. Dis..

[17] World Health Organization (2021). COVID-19 Clinical Management: Living Guidance.

[18] Clinical management of severe acute respiratory infection when novel coronavirus (nCoV) infection is suspected. WHO Interim guidance. https://apps.who.int/iris/bitstream/handle/10665/332299/WHO-2019-nCoV-Clinical-2020.1-eng.pdf.

[19] Barasa E., Kairu A., Ng’ang’a W., Maritim M., Were V., Akech S., Mwangangi M. (2021). Examining unit costs for COVID-19 case management in Kenya. BMJ Global Health. BMJ Glob. Health.

[20] Home Care for Patients with Suspected or Confirmed COVID-19 and Management of Their Contacts. WHO Interim Guidance. https://apps.who.int/iris/bitstream/handle/10665/333782/WHO-2019-nCoV-IPC-HomeCare-2020.4-eng.pdf.

[21] Zhou F., Yu T., Du R., Fan G., Liu Y., Liu Z., Xiang J., Wang Y., Song B., Gu X. (2020). Clinical course and risk factors for mortality of adult inpatients with COVID-19 in Wuhan, China: A retrospective cohort study. Lancet.

[22] Huang C., Wang Y., Li X., Ren L., Zhao J., Hu Y., Zhang L., Fan G., Xu J., Gu X. (2020). Clinical features of patients infected with 2019 novel coronavirus in Wuhan, China. Lancet.

[23] Driouich J.-S., Cochin M., Lingas G., Moureau G., Touret F., Petit P.-R., Piorkowski G., Barthélémy K., Laprie C., Coutard B. (2021). Favipiravir antiviral efficacy against SARS-CoV-2 in a hamster model. Nat. Commun..

[24] Bosaeed M., Alharbi A., Hussein M., Abalkhail M., Sultana K., Musattat A., Alqahtani H., Alshamrani M., Mahmoud E., Alothman A. (2021). Multicentre randomised double-blinded placebo-controlled trial of favipiravir in adults with mild COVID-19. BMJ Open.

[25] Agrawal U., Raju R., Udwadia Z.F. (2020). Favipiravir: A new and emerging antiviral option in COVID-19. Med. J. Armed. Forces India.

[26] Fact Sheet for Health Care Providers Emergency Use Authorization (EUA) of Bamlanivimab. https://www.fda.gov/media/143603/download.

[27] Lundgren J.D., Grund B., Barkauskas C.E., Holland T.L., Gottlieb R.L., Sandkovsky U., Brown S.M., Knowlton K.U., Self W.H., ACTIV-3/TICO LY-CoV555 Study Group (2021). A Neutralizing Monoclonal Antibody for Hospitalized Patients with COVID-19. N. Engl. J. Med..

[28] Gottlieb R.L., Nirula A., Chen P., Boscia J., Heller B., Morris J., Huhn G., Cardona J., Mocherla B., Stosor V. (2021). Effect of Bamlanivimab as Monotherapy or in Combination with Etesevimab on Viral Load in Patients with Mild to Moderate COVID-19: A Randomized Clinical Trial. JAMA.

[29] Tjordan_Drupal. FDA Revokes Emergency Use Authorization for Monoclonal Antibody Bamlanivimab|AHA News. https://www.aha.org/news/headline/2021-04-19-fda-revokes-emergency-use-authorization-monoclonal-antibody-bamlanivimab.

[30] Taylor P.C., Adams A.C., Hufford M.M., de la Torre I., Winthrop K., Gottlieb R.L. (2021). Neutralizing monoclonal antibodies for treatment of COVID-19. Nat. Rev. Immunol..

[31] Evaluation of COVID-19 Vaccine Effectiveness. https://www.who.int/publications-detail-redirect/WHO-2019-nCoV-vaccine_effectiveness-measurement-2021.1.

[32] Biccard B.M., Gopalan P.D., Miller M., Michell W.L., Thomson D., Ademuyiwa A., Aniteye E., Calligaro G., Chaibou M.S., Dhufera H.T. (2021). Patient care and clinical outcomes for patients with COVID-19 infection admitted to African high-care or intensive care units (ACCCOS): A multicentre, prospective, observational cohort study. Lancet.

[33] Corti D., Purcell L.A., Snell G., Veesler D. (2021). Tackling COVID-19 with neutralizing monoclonal antibodies. Cell.

